# Take Care in the Kitchen: Avoiding Cooking-Related Pollutants

**DOI:** 10.1289/ehp.122-A154

**Published:** 2014-06-01

**Authors:** Nate Seltenrich

**Affiliations:** Nate Seltenrich covers science and the environment from Petaluma, CA. His work has appeared in *High Country News*, *Sierra*, *Yale Environment 360*, *Earth Island Journal,* and other regional and national publications.

Carbon monoxide (CO), nitrogen dioxide (NO_2_), and particulate matter (PM) are harmful air pollutants that pose significant short- and long-term health risks. Emitted from coal-fired power plants, vehicle exhaust pipes, and other combustion sources, they’re among six primary pollutants monitored by the U.S. Environmental Protection Agency (EPA) through the Clean Air Act.^1^ These same pollutants are also some of the most common contributors to unhealthy air inside U.S. homes, due in part to a ubiquitous and possibly surprising activity: cooking.^2^^,^^3^^,^^4^

**Figure:**
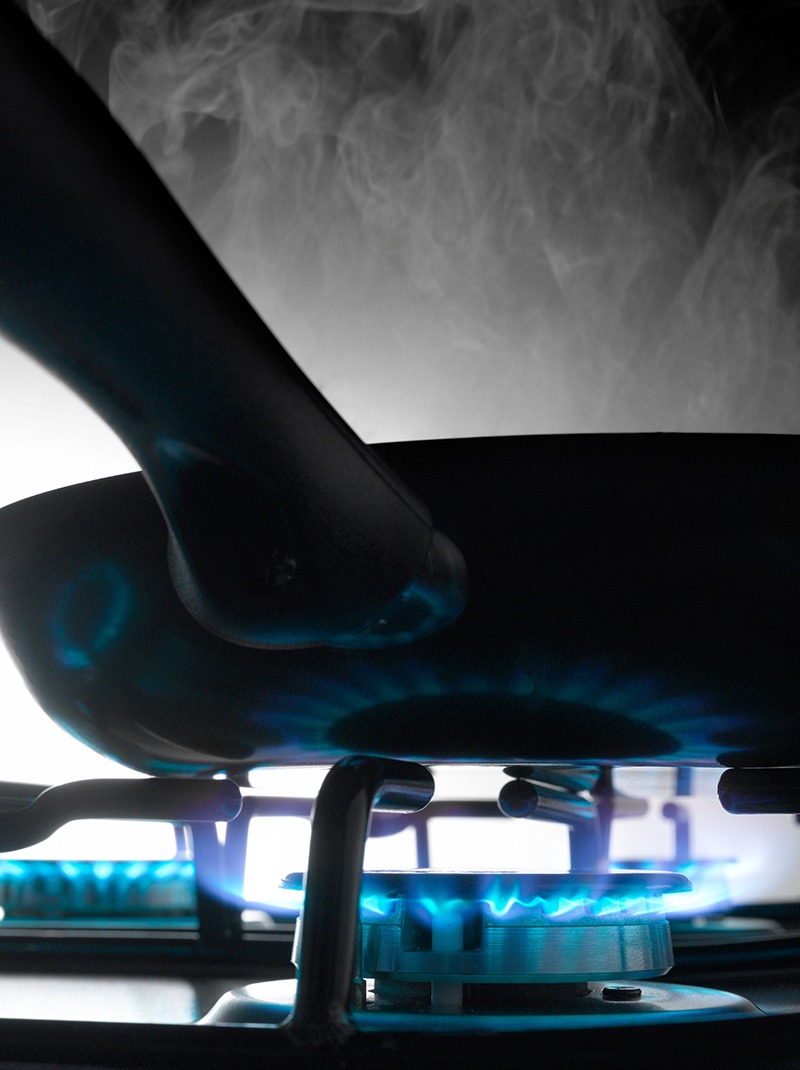
Cooking can release potentially hazardous pollutants, but taking a few simple steps reduces exposures. © Photocuisine/Alamy

Researchers now understand that the process of cooking food and even simply operating stoves—particularly gas appliances—can emit a cocktail of potentially hazardous chemicals and compounds. Within our homes, these pollutants are less diluted than they are outdoors, and in the absence of proper ventilation, they often are trapped inside.^5^ The World Health Organization has established general guidelines for indoor air quality^6^ and is currently developing specific limits related to burning solid fuels for cooking and heating. However, indoor air in nonindustrial buildings is not regulated by the EPA or any other U.S. agency.

“Literally millions to many millions of people are routinely being exposed to air pollutants at levels that we don’t allow outdoors,” says Brett Singer, a staff scientist at Lawrence Berkeley National Laboratory (LBNL) who studies indoor air quality and cooking emissions in particular. His team modeled gas stove emissions and exposures in California households and estimated that during a typical winter week—when windows are more likely to be closed and air exchange lower—1.7 million Californians could be exposed to CO levels that exceed national and state ambient air quality standards, simply by cooking on gas stoves without the use of a range hood. Twelve million could be exposed to excessive levels of NO_2_.^7^

“That’s a lot of people in California, and those results ballpark-apply across the country,” Singer says. “The EPA would say we don’t have a carbon monoxide or nitrogen dioxide problem in this country,” he says, since average outdoor concentrations nationwide fall well below the agency’s safety standards.^8^^,^^9^ “In reality,” he adds, “we absolutely do have that problem; it’s just happening indoors.”

The solution offered by experts is certainly not to stop cooking. Rather, improved ventilation and filtration, achieved through better-designed range hoods and more robust building codes and standards, could ensure occupant safety by removing these pollutants from indoor air.^10^ In the meantime, experts recommend a few simple strategies to reduce exposures.

## How Important Are Cooking Pollutants?

Attention to indoor air quality began to grow after the oil crisis of the 1970s, which set in motion a widespread effort among homeowners and builders to seal up leaky houses to improve energy efficiency, says Max Sherman, a member of the Berkeley Lab Residential Building Systems team. But in the absence of well-designed ventilation, tightening homes can also mean trapping pollutants from a variety of interior sources, including furniture, carpet, paint, heaters, and kitchen appliances, posing elevated health risks to occupants. The smaller and more airtight the home, the greater the risk.

**Figure:**
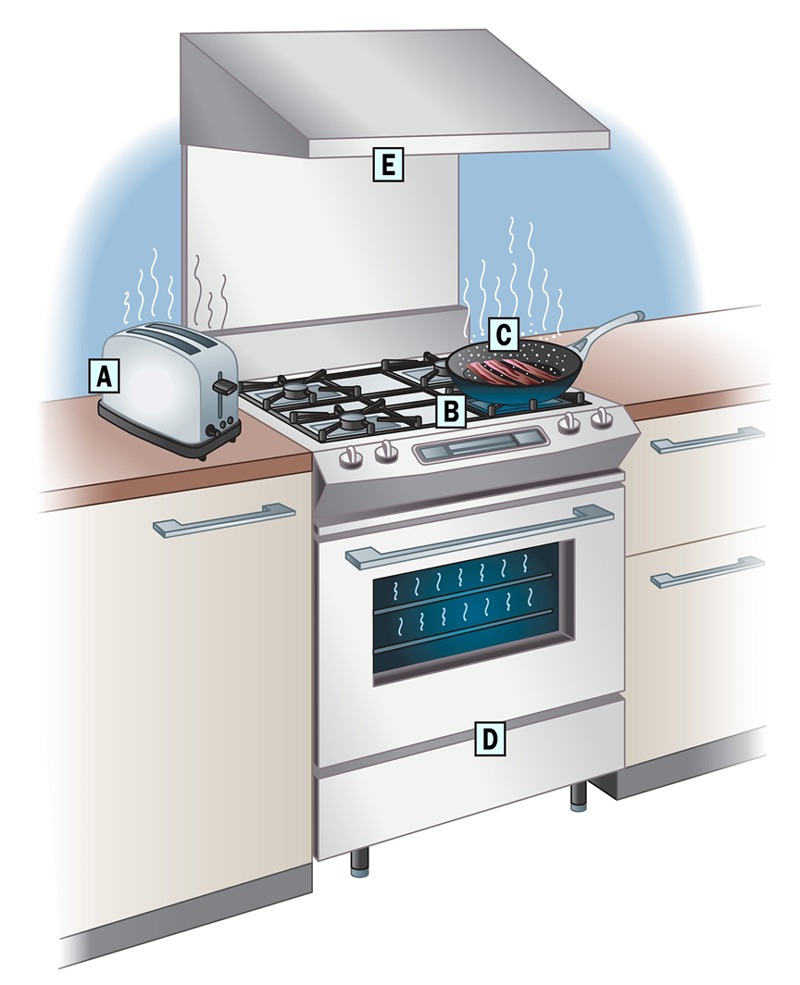
Cooking appliances and the process of cooking itself can produce numerous pollutants. For instance, electric coil burners in stoves, ovens, and toasters (A) can release fine and ultrafine particles,^25^^,^^26^ while gas burners (B) can generate nitrogen dioxide, carbon monoxide, particulate matter, and formaldehyde.^26^^,^^27^ The burning of organic matter during cooking (C)—particularly with high-temperature activities like frying, broiling, and sautéing—produces acrolein,^28^ polycyclic aromatic hydrocarbons,^29^ and particulate matter.^26^ An appliance’s pilot light (D) can be a source of nitrogen dioxide.^20^ A venting hood (E), even if only modestly effective, can protect residents against cooking-related pollutants.^21^ © 2014 Daniel Gallant

“I realized in the late 1980s that we were on a track where if we kept tightening things up for energy reasons, we weren’t going to get enough air for ventilation reasons, so we were going to need to define minimum ventilation standards,” Sherman says. In 1997 the American Society of Heating, Refrigerating and Air Conditioning Engineers (ASHRAE) created a committee to develop a ventilation standard for single-family homes in the United States. Sherman was appointed chairman.

The first version of its ventilation code, known as ASHRAE 62.2,^11^ took effect in 2003. Among its requirements for new homes was kitchen ventilation capable of exhausting pollutants to the outdoors, typically through the use of an overhead range hood. The code also stipulated a minimum airflow level and maximum noise level for range hoods.

Many municipalities were slow to embrace the standard, however, and today only a few states have formally adopted ASHRAE 62.2 as part of their building code, says Iain Walker, a staff scientist on the LBNL team. Others have integrated it into broader energy and weatherization codes that apply to some but not all new homes. According to Walker, a confusing array of local, state, and international building codes (on which many domestic codes are based) contain differing kitchen ventilation standards, if they require them at all.

Walker estimates that about half of all new homes built in the United States today comply with ASHRAE 62.2 guidelines. This figure is on an upward trend, he says, as new homes become more energy-efficient and airtight, making ventilation an increasingly important consideration in homebuilding.

Statistics on kitchen ventilation across the entire U.S. housing stock are harder to come by. In 2011 Singer and colleagues explored a novel method for collecting such data. They examined listing photographs of 1,002 California homes for sale on a real estate website to see what type of hoods were pictured in the kitchens. They found that 47% had a combination overhead microwave/range hood installed, a higher percentage than for any other single hood type.^12^ According to Singer, no existing combination microwave/range hoods meet ASHRAE 62.2’s airflow and noise-level limits.

Some kitchens have “ductless” hoods that recirculate fumes through activated charcoal filters rather than exhaust them outdoors, which Walker says does very little to clean the air. Ductless hoods are cheaper than venting hoods because they do not require the installation of ducts or vents, although the filters must be replaced regularly. New homes in areas where ASHRAE 62.2 does not apply often feature recirculating hoods by default, Walker says.

Even the best range hoods are useless until switched on. Survey data suggest that excessive noise produced by most fans discourages people from using them regularly.^13^

Four decades after scientists first recognized indoor air quality and gas-stove emissions as a health concern in many U.S. homes, relatively few people think of unvented cooking as a potentially hazardous activity, Sherman says. “People have been cooking since the dawn of time, so it’s considered a very normal activity,” he says. “I think in the last year or two, there’s been some research that has gotten people a little more concerned about it ... but I wouldn’t say it’s a high level of concern in the population.”

The LBNL team is working both science and policy angles to address the issue.^14^ This includes running experiments to better understand the type, quantity, and epidemiology of cooking contaminants, as well as participating in policy discussions to encourage strengthening and broader adoption of ASHRAE 62.2. They also are communicating with manufacturers to help develop quieter, more effective, affordable range hoods that can switch on automatically. Finally, they’re leading an effort to develop a new “capture-efficiency” metric for residential range hoods that will provide consumers a reliable indication of real-world performance.

In order to move their work from the lab to people’s kitchens, Walker believes researchers must develop a better grasp on what pollutants are emitted during cooking, at what levels, and what the pollutants’ potential health impacts are. “We need to be able to say, ‘What’s the risk if we don’t ventilate right?’ before we can make all these changes,” he says.

## Assessing the Health Impacts

Part of the challenge in assessing the health effects of cooking pollutants is that levels and exposures can vary widely depending on the stove, cooking activity and temperature, burner location, and ventilation level, says Singer. Even in controlled laboratory experiments, tiny variations in cooking behavior can produce significant changes in particle emissions. He explains, “These activities are fundamentally variable, even when they seem the same. When people are cooking, it’s hard to predict the things that will produce the most pollutants.”

The health effects of some cooking pollutants are relatively well understood. For instance, studies have demonstrated associations between elevated indoor levels of NO_2_, often attributable directly to gas stove burners, and symptoms in children including chest tightness, shortness of breath, wheeze, and increased asthma attack frequency.^15^^,^^16^^,^^17^^,^^18^

Yet the full nature and scope of pollutants produced by modern-day cooking remains unclear, let alone the extent to which cooking-pollutant exposures may affect occupants’ health. In 2012 the LBNL group published a method for estimating the chronic health impact of dozens of air pollutants found in U.S. homes, including common cooking-related pollutants.^19^ They estimated that the cumulative health burden of the pollutants studied amounted to between 400 and 1,100 disability-adjusted life-years lost annually per 100,000 persons.

Of note to cooking-pollutant research, PM_2.5_, acrolein, and formaldehyde were responsible for the majority of the impacts.^19^ All three are produced by gas stoves or cooking activities—among other potential indoor sources—although the authors did not estimate how much individual sources contributed to health burden.

Lead author Jenny Logue says, “If everybody cooks with no range hood, what does that mean in terms of cardiac arrest, and what does that mean in terms of how many people go to the hospital?” Investigators still aren’t sure.

Brian Leaderer, a professor of epidemiology at Yale University who studies the health effects of NO_2_, particularly in children, believes a next step in this line of investigation will be to perform double-blind intervention studies to determine if health improvements follow the reduction or removal of cooking-pollutant exposures. “I think the significant population at risk would be in major cities where gas lines are densest, where gas stoves are most prominent, and especially in multi-home housing projects where the size of the home tends to be smaller and the [pollutant] concentration tends to be higher,” he says.

Leaderer adds that inner cities also tend to have high asthma rates. “You have a sensitive population exposed to the highest NO_2_ concentrations, and this should be a health target population to reduce exposures and see if the benefits are there,” he says.

Nadia Hansel, a researcher, professor, and medical doctor with Johns Hopkins Medicine, has also studied exposures and health effects of NO_2_ from gas stoves, particularly in relation to asthma and chronic obstructive pulmonary disease (COPD). In one study, she found that swapping a gas stove for an electric stove was associated with a 51% decrease in median kitchen NO_2_ concentrations after 3 months, while use of a portable air purifier with HEPA and activated carbon filters was associated with a 19% decrease in median kitchen NO_2_ concentrations.^20^

Again, however, it’s unclear if those reductions can be tied directly to healthier occupants. “For patients that have chronic underlying lung disease, it is potentially quite important,” Hansel says. “If you can reduce exposure for those people, you might reduce symptoms and improve their health.”

Even among the broader population, including healthy adults, she says, cooking pollutants in combination with outdoor air pollutants could potentially contribute to loss of lung function and development of COPD and asthma. “It’s quite possible that it could lead to problems with chronic lung disease,” she says. “The data’s out there suggesting [the impact of exposure] can be cumulative over time.”

Less epidemiological evidence exists around the health effects of ultrafine particles in indoor air, of which cooking is the primary source, says Lance Wallace, a former EPA scientist and now a guest researcher with the National Institute of Standards and Technology. Based on available information, he believes that ultrafine particles are at least as toxic as fine particles, “and fine particulates have a long history of causing morbidity and mortality in this country and in developing countries,” he says.

## Avoiding Exposures

In tandem with ongoing health research, the LBNL team aims to better understand the performance—and shortcomings—of existing range hoods. “If we can vent everything, then we don’t have to worry about what the health impacts are,” says Walker.

**Figure:**
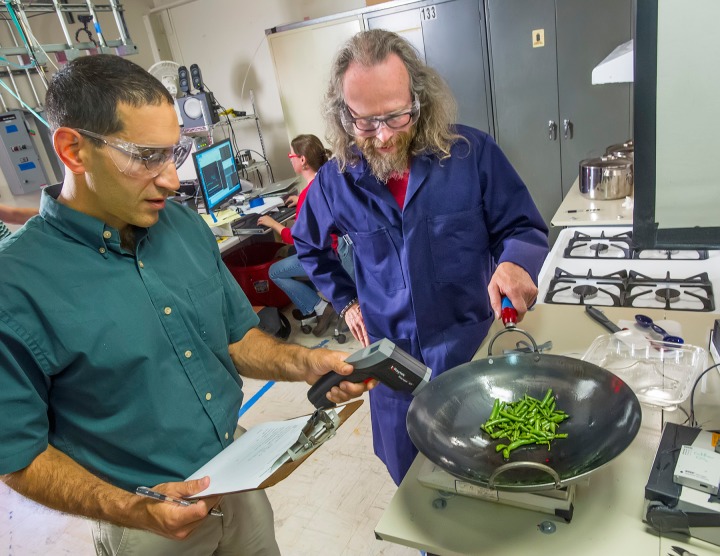
Tiny variations in cooking can dramatically affect emissions, so when researchers Brett Singer (left) and Woody Delp (right) study cooking-related pollutants, they take care to replicate temperature, fuel flow, cooking time, and other factors. Here, in their LBNL lab, Singer uses an infrared thermometer to ensure the temperature of the pan is the same during each standardized cooking event. © 2013 The Regents of the University of California, Lawrence Berkeley National Laboratory

Inside a small laboratory on the LBNL campus, Singer and colleague Woody Delp designed a mock kitchen to generate, vent, and measure pollutants associated with gas stoves and cooking processes. Above a four-burner stove is a range-hood mount allowing different devices to be easily secured to a six-inch duct. The duct, in turn, is equipped with a sensor to measure concentrations of pollutants within the exhaust air. On the floor nearby is a motley assemblage of range hoods. Singer and Delp used this setup to rate residential cooking exhaust devices for a recent study^21^ (a similar study^22^ was conducted in actual homes).

To compare the hoods, the investigators relied on capture efficiency. This metric is calculated by sampling pollutant levels inside the test kitchen after using the stove without running the hood and comparing them to pollutant levels in the ducts after using the stove with the hood switched on. A capture efficiency of 50% means half the pollutants generated during cooking were captured by the hood.

The appliances exhibited a wide range of abilities, although most fell toward the low end of the performance scale. The lab study, which examined seven hoods representing a cross-section of exhaust devices available to U.S. consumers, found capture efficiencies from less than 15% to greater than 98%, depending on both the hood tested and the burners used (front versus rear). Capture efficiencies were significantly higher for rear burners because wall-mounted hoods typically do not extend all the way over front burners. Additionally, devices with open, scoop-like capture hoods performed considerably better than flat-bottomed exhaust devices such as typical combination microwave/range hoods.^21^

The hood with the highest average capture efficiency in the test (greater than 80%) had a large scoop that extended most of the way over the front burners.^21^ Yet its airflow rate exceeded industry-recommended levels. Excessive airflow in range hoods can quickly depressurize airtight, energy-efficient homes, Singer says. This can draw in outdoor air through any available opening, or cause exhaust gases from other interior combustion appliances (for instance, furnaces and natural-draft water heaters) to fail to properly exhaust to the outside.

The highest-performing model also produced sound levels too high for normal conversation, an inconvenience likely to result in less frequent use. Among hoods meeting sound-level and energy-consumption criteria set by the ENERGY STAR_®_ ratings system, capture efficiency was less than 30% for front and oven burners. In other words, these code-compliant and energy-efficient exhaust devices captured, on average, less than a third of the pollutants emitted during cooking.^21^

The in-home study, which evaluated a convenience sample of 15 different devices, yielded similar conclusions.^22^ Singer and Delp’s initial findings have been replicated by other recent studies showing the limited effectiveness of range hoods at exhausting ultrafine particles emitted during cooking.^23^^,^^24^

That said, some ventilation is better than none. Even moderately effective venting range hoods, used regularly, can substantially reduce exposures to cooking-related pollutants, according to Singer and Delp. They also recommend cooking on the back burners whenever possible (they tend to be vented more completely) and using higher fan settings.^21^

## Toward Better Hoods

To encourage the development of better range hoods, the LBNL team is working with other researchers and several major appliance manufacturers to design a capture-efficiency test method for universal use. Ultimately, they hope, capture efficiency will replace airflow as the most prominent performance metric printed on range-hood labels and as the basis for ENERGY STAR ratings.

“Basically, the push in the industry is to … get at a more intelligent design that really accomplishes what the consumer is looking to do, which is remove the pollutants from the space in a potentially more energy-efficient manner,” says Mike Moore, a consultant who represents appliance manufacturers on building performance and environmental quality issues.

Sherman says a capture-efficiency threshold is being considered for inclusion in the next update of ASHRAE 62.2 (Singer and Walker suggest a lower limit of 80%), meaning that within a few years the code itself could move from merely requiring mechanical kitchen ventilation to demanding range hoods that perform well. “Just having any old hood doesn’t tell you how good that hood is at capturing what you actually want to capture,” Sherman says.

Another hood element likely to be included in the next version of ASHRAE 62.2, whether as a requirement or as a recommendation, is the use of auto-on features triggered by light sensors, heat sensors, or wireless signals between the range and the hood, says Michael Lubliner, a building science specialist with Washington State University. He says these features, currently available on a few high-end models, could improve the devices’ effectiveness across the board. In addition, investigators suggest that kitchen exhaust devices could eventually be outfitted with HEPA and carbon filters to handle NO_2_ and particulate matter.

Ultimately, what’s most important is to develop an affordable, automatic range hood that’s quiet and effective. “That’s where we need to end up as an industry,” Walker says. “It’s all doable, and it’s not complicated.”
