# Erratum: “Take Care in the Kitchen: Avoiding Cooking-Related Pollutants“

**DOI:** 10.1289/ehp.123-A202

**Published:** 2015-08-01

Seltenrich N. 2014. Take care in the kitchen: avoiding cooking-related pollutants. Environ Health Perspect 122(6):A154–A159; http://dx.doi.org/10.1289/ehp.122-A154

In the original version of this article, the National Institute of Standards and Technology was incorrectly called the National Institute of Science and Technology.

*EHP* regrets the error.

